# ‘Silver’ trauma: predicting mortality in elderly major trauma based on place of injury

**DOI:** 10.1186/1757-7241-23-S2-A4

**Published:** 2015-09-11

**Authors:** Susan A Hendrickson, Denise Osei-Kuffour, Christopher Aylwin, Michael Fertleman, Shehan Hettiaratchy

**Affiliations:** 1 Major Trauma Centre, St. Mary's Hospital, Imperial College Healthcare NHS Trust, London, UK

## Background

Elderly trauma now accounts for more than 20% of UK major trauma [1]. Clearly, elderly trauma patients are at increased risk of morbidity and mortality, but outcome can be difficult to predict. Current evidence is limited to correlations between poor outcome and complex, difficult-to-calculate pre-morbid frailty indices [2]. A simple, evidence-based prognostic marker is needed to guide early management. We hypothesised that elderly patients suffering severe injury at home indoors are frailer, and therefore more likely to succumb to their injuries, than those injured outdoors.

## Method

All patients admitted to a London major trauma centre in 2013 aged ≥65 years old with an Injury Severity Score (ISS) >15 were identified using Trauma Audit & Research Network data. Patient demographics, date of death, and injury location (‘at home indoors’ or ‘outside the home’) were recorded and mortality rates compared.

## Results

124 patients were included (M:F = 1.4:1). 58 patients (46.8%) were injured at home indoors; 66 patients (53.2%) outside the home. The groups were equivalent in age (p=0.44) and ISS (p=0.52). 6-month mortality among patients injured at home indoors was 36.2%, nearly double that of patients injured outside of the home (18.2%) (p=0.0267).

## Conclusion

Our study found significant correlation between injury location and mortality, suggesting that severe injury inside the home could represent a rough marker of frailty, and therefore, outcome. This could have important implications for prognosticating elderly patients in the resuscitation room, where quick management decisions need to be made by clinicians, patients and relatives. Further studies are needed to evaluate pre-morbid frailty of both groups and to assess functional outcome of survivors.
